# Differentiation of bilateral BI-RADS 4 breast lesions with one benign and one malignant finding using dual-layer detector spectral CT: a case report

**DOI:** 10.3389/fonc.2026.1771427

**Published:** 2026-04-01

**Authors:** Ying Chen, Qi Wang, Qian Xu, Xiangyao Gong, Jing Wen, Lili Zhou, Yu Du

**Affiliations:** 1 Department of CT and MRI, The Fourth Hospital of Hebei Medical University, Shijiazhuang, China; 2 Philips (China) Investment Co., Ltd., Beijing Branch, Beijing, China

**Keywords:** benign-malignant differentiation, BI-RADS 4, breast lesions, quantitative imaging, spectral CT

## Abstract

The differentiation between benign and malignant BI-RADS 4 breast lesions remains a significant clinical challenge, particularly when multi-modal imaging assessments yield inconsistent results, often leading to diagnostic dilemmas. Dual-layer detector spectral computed tomography (CT) (DLCT), as an imaging modality, provides multi-parametric quantitative information and holds promise as a tool for lesion differentiation. Here, we present a case of bilateral breast lesions. Although ultrasound assessed both as BI-RADS 4, subsequent mammography, magnetic resonance imaging (MRI), and CT yielded discordant findings for the right lesion. Retrospective analysis of the preoperative DLCT data clearly revealed the intrinsic differences between the bilateral lesions. This accurately predicted malignancy on the left side and benignity on the right side, which was in complete concordance with the final surgical pathological findings.

## Introduction

Breast cancer is the most common malignant tumor among women and one of the leading causes of cancer-related mortality in females (1, 2). Early diagnosis and treatment can significantly improve survival rates and quality of life. The Breast Imaging Reporting and Data System (BI-RADS) provides a standardized and structured approach to evaluating breast lesions. However, the malignancy risk of BI-RADS 4 lesions ranges from 2% to 95%, representing a wide span, and the differentiation between benign and malignant lesions remains a major clinical challenge. Typically, biopsy or surgical excision is recommended to clarify the pathological nature of the lesion, which may result in unnecessary procedures and psychological burden for some patients (3). Therefore, non-invasive and accurate differentiation of malignancy in BI-RADS 4 lesions is a current research focus.

Dual-layer detector spectral computed tomography (DLCT), as a new imaging technique combining both functional and molecular imaging, provides multi-parametric quantitative information through the post-processing of a single low-dose scan. Increasing studies have investigated its diagnostic value in breast diseases, with quantitative parameters showing significant potential for distinguishing benign from malignant lesions. For instance, Demirler et al. (4) demonstrated that quantitative parameters—including iodine content (IC), effective atomic number (Zeff), and spectral curve slope (λHu)—offer superior diagnostic performance in differentiating benign from malignant breast tumors, with an optimal iodine threshold of 0.7–0.9 mg/mL (sensitivity: 96.6%, specificity: 91.7%). Similarly, Wang et al. (5) found that malignant lesions had significantly higher normalized iodine concentration (NIC), λHu, normalized effective atomic number (NZeff), and CT values during both arterial and venous phases than benign lesions, with venous phase λHu yielding the best diagnostic performance (AUC:0.9, sensitivity: 84.1%, specificity: 86.3%, accuracy: 85.7%). More recently, the extracellular volume fraction (ECV) derived from spectral CT has been investigated as a biomarker of the tumor microenvironment, with Jiang et al. (6) reporting significant ECV differences between malignant and benign lesions. It provides a novel, multi-dimensional method for the differential diagnosis of breast lesions.

Although previous studies have confirmed the value of spectral CT in differentiating breast lesions, these investigations have primarily focused on lesions that are evaluable on conventional imaging. The application value of spectral CT for BI-RADS category 4 lesions with discordant findings on multimodality imaging remains to be explored. This report aims to retrospectively explore the value of spectral CT in the differential diagnosis of BI-RADS 4 breast lesions through a case in which both bilateral breast lesions were assessed as BI-RADS 4 on ultrasound, but the final pathology revealed one malignant and one benign lesion.

## Case presentation

**Figure 1:**
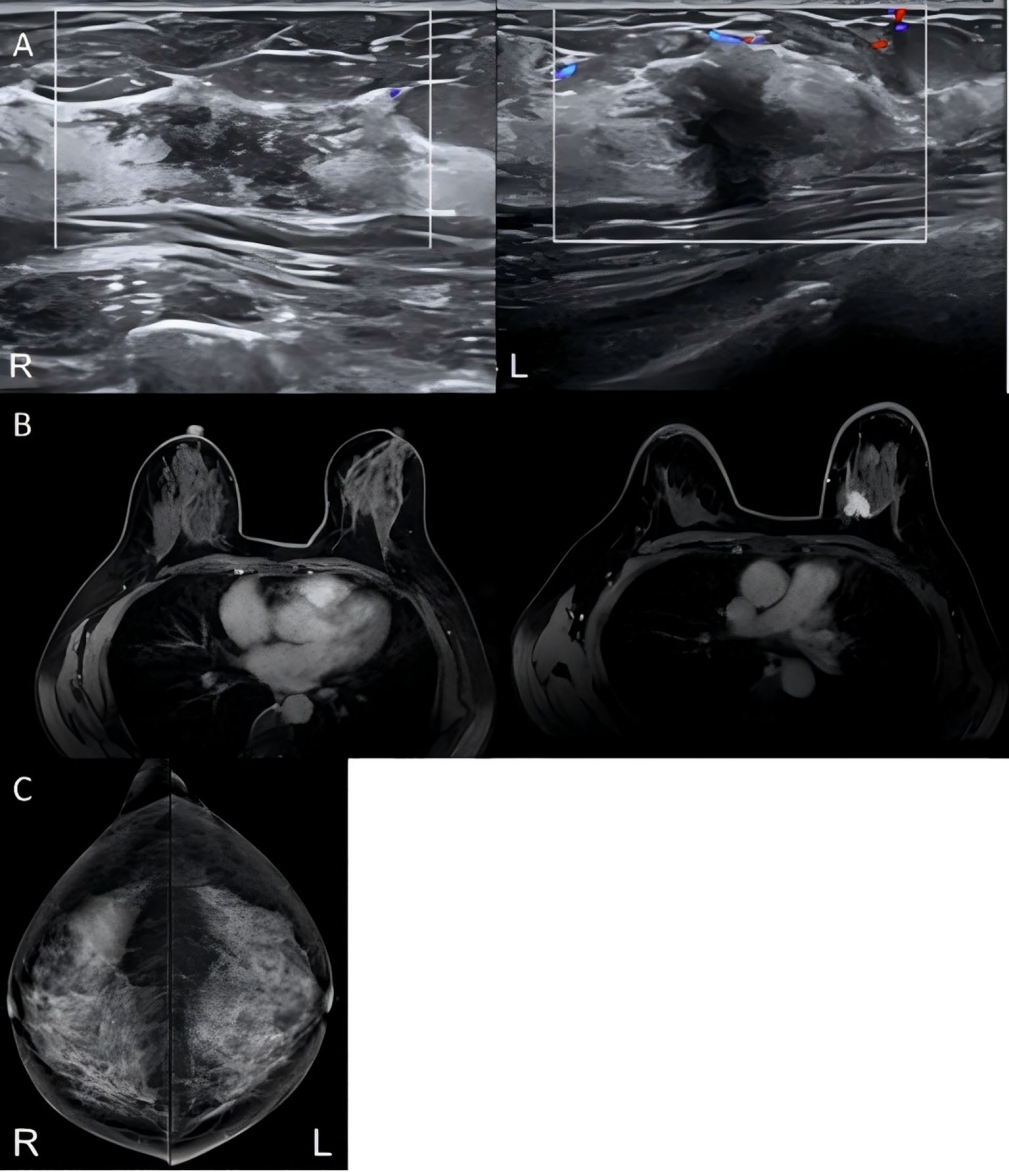
Conventional imaging findings of the patient. **(A)** Ultrasound: right breast shows an irregular sheet-like hypoechoic area with ill-defined borders and scant internal vascularity; left breast shows an irregular hypoechoic nodule with ill-defined borders, spiculated margins, and peripheral vascularity. **(B)** Contrast-enhanced MRI: left breast shows a well-defined, homogeneously enhancing mass in the upper inner quadrant; right breast is unremarkable with no abnormal enhancement. **(C)** Mammography: left breast shows an irregular isodense mass with spiculated margins in a heterogeneously dense background; right breast demonstrates no definite mass or suspicious calcifications.

A 49-year-old female with a left breast lump detected during routine physical examination was referred to our Breast Department for further management. The lump was without skin erythema, ulceration, nipple discharge, or breast tenderness. The patient denied any significant prior medical conditions or family history of cancer. Menarche occurred at age 13 (regular cycles, moderate flow), and she experienced menopause at age 48. The patient married at 29 and is gravida 1, para 1, having had one full-term delivery of a healthy child. Since symptom onset, her general condition has been unremarkable. All imaging studies were conducted at our institution. Breast ultrasound was performed and preliminarily interpreted by an experienced attending sonologist and subsequently confirmed by a deputy chief sonologist. Mammography and magnetic resonance imaging (MRI) images were independently and blindly evaluated by two radiologists (associate senior titles); any disagreements were resolved by consensus. All imaging assessments were strictly performed in accordance with the BI-RADS. Ultrasound examination revealed a solid hypoechoic nodule in the left breast with irregular shape, unclear boundaries, spiculated margins, and enhanced surrounding tissue echogenicity, with blood flow signals detected around the lesion. The right breast showed an irregular, hypoechoic, sheet-like area with unclear boundaries, containing numerous small punctate hyperechoic foci and minimal blood flow signals. Both lesions were classified as BI-RADS 4 (right: 4B;left: 4C) (**Figure 1A**). Breast magnetic resonance imaging (MRI) with contrast enhancement demonstrated dense glandular tissue in both breasts. In the upper inner quadrant of the left breast, a well-defined, homogeneously enhancing mass was observed (BI-RADS 6), while no significant abnormalities were noted in other areas (**Figure 1B**). Mammography revealed that both breasts were dense, with some glandular tissue showing sheet-like high-density areas. An irregular mass of equal density with spiculated margins was observed in the left breast (BI-RADS 6), while the right breast showed no obvious mass or suspicious calcifications (BI-RADS 2) (**Figure 1C**).

**Figure 2:**
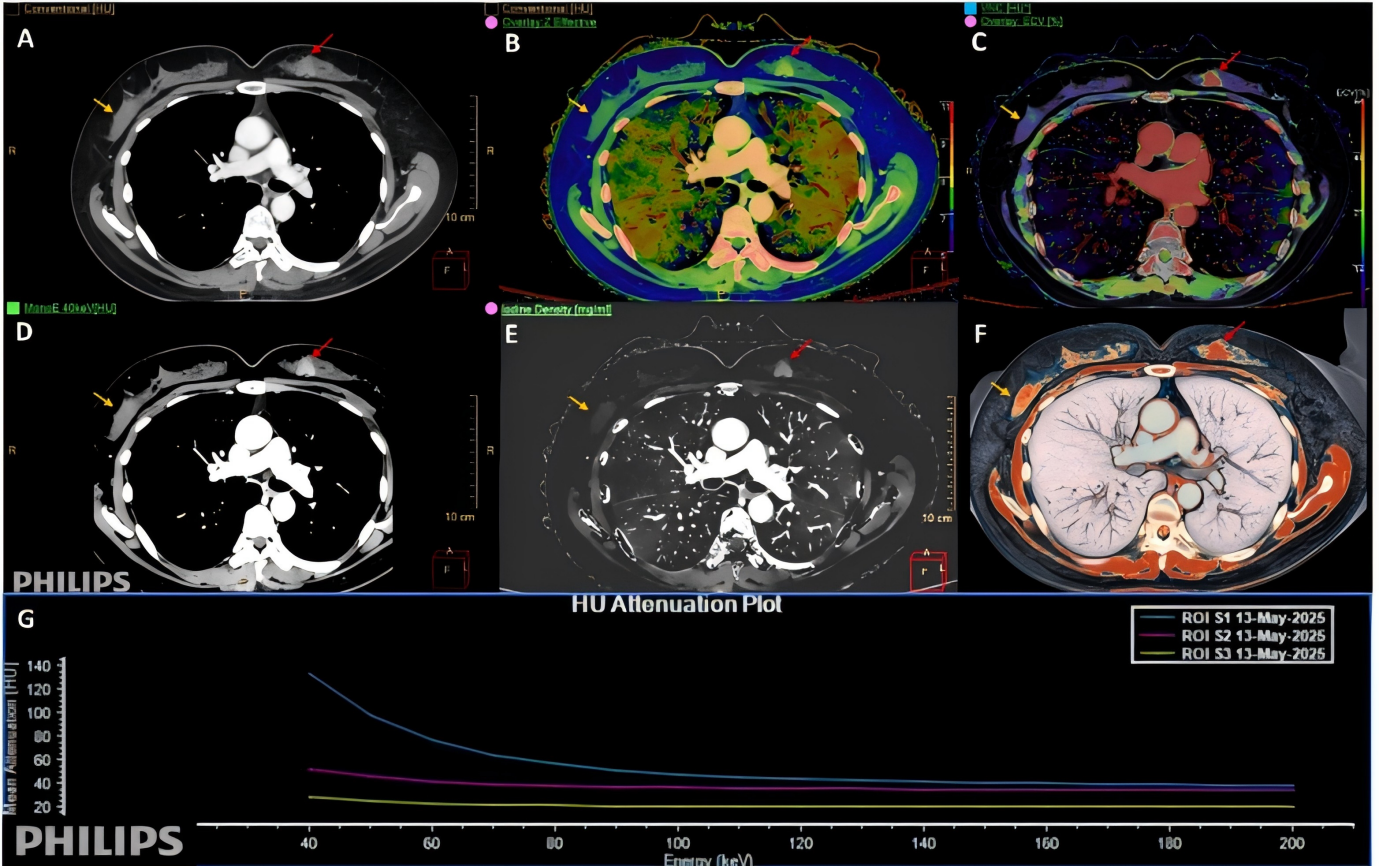
Multi-parametric DLCT analysis of the patient. **(A)** Conventional CT image, **(B)** Effective atomic number map, **(C)** ECV map, **(D)** 40 keV virtual monoenergetic image, **(E)** iodine density map, **(F)** PRVR biomimetic image, **(G)** spectral curves. Red arrows indicate the malignant lesion in the left breast; yellow arrows indicate the benign lesion in the right breast. Spectral curves: left lesion (S1, blue) shows a steep descending slope; right lesion (S2, purple) shows a flat slope, approximately parallel to normal glandular tissue (S3, yellow).

The patient also underwent contrast-enhanced DLCT. The spectral data were transferred to the Philips IntelliSpace Portal post-processing workstation. Conventional CT images, virtual monoenergetic images, iodine density maps, effective atomic number maps, extracellular volume (ECV) maps, and Photo Realistic Volume Rendering (PRVR) biomimetic images (**Figure 2**) were generated using this workstation. Two radiologists (with 2 and 10 years of experience in breast imaging), blinded to clinical information and final pathology, independently placed regions of interest (ROIs). ROIs were initially drawn on venous phase 40 keV VMI to encompass the enhancing portions of the bilateral lesions while carefully avoiding cystic, necrotic, or calcified areas. The ROIs were then semi-automatically copied to the venous phase images of the conventional CT image, the iodine density, Zefective map and the ECV maps to ensure consistent size, shape, and position between the different images. Each ROI measurement was performed three times, and the average value was calculated for subsequent analysis.

Conventional CT images in venous phase showed a poorly defined soft tissue nodule with mild enhancement in the upper inner quadrant of the left breast. In contrast, no distinct mass was observed in the dense glandular tissue of the right breast (**Figure 2A**). DLCT multi-parametric analysis revealed significant differences between the two lesions: On the 40 keV virtual monoenergetic images, the left breast lesion showed focal, marked enhancement with clear borders, with a CT value of 133.2 HU, while the right breast lesion demonstrated only mild enhancement with a CT value of 52.2 HU, with a blurry boundary compared to surrounding glandular tissue (**Figure 2D**). The iodine density map showed clear high iodine uptake in the left breast lesion, with an iodine concentration of 1.15 mg/ml, whereas the right breast lesion exhibited diffusely low iodine uptake with an iodine concentration of 0.22 mg/ml (**Figure 2E**). The effective atomic number map revealed a value of 8.02 for the left breast lesion and 7.40 for the right breast lesion (**Figure 2B**), reflecting differences in the material composition between the two lesions. Extracellular volume mapping showed an ECV of 57% for the left breast lesion and 11.8% for the right breast lesion (**Figure 2C**), indicating differing extracellular matrix distribution patterns. The PRVR biomimetic images significantly enhanced the visualization of the lesions (**Figure 2F**). Further analysis of the spectral curves drawn from the regions of interest (ROIs) in the bilateral lesions (S1 for the left breast and S2 for the right breast) and normal glandular tissue (S3) revealed that the spectral curves of S1 and S2 were not consistent, suggesting different origins, while the curves for S2 and S3 were nearly parallel, indicating similar biological activity (**Figure 2G**). Based on these spectral parameters, the left breast lesion (S1) showed high iodine uptake, high ECV, high effective atomic number, and a steep spectral curve, consistent with malignant characteristics. The right breast lesion (S2) demonstrated extremely low iodine uptake, low effective atomic number, low ECV, and a flat spectral curve, resembling the normal glandular tissue and indicative of benign features.

The biopsy pathology revealed invasive carcinoma grade II in the left breast and fibrous tissue hyperplasia in the right breast. Given the clear malignancy of the left breast lesion and the uncertain nature of the right lesion, the patient underwent segmental resection of the left breast glandular tissue and excision of the right breast mass. Postoperative pathology of the specimens showed: invasive ductal carcinoma with invasive ductal carcinoma *in situ* and lobular carcinoma *in situ* in the left breast; and extensive fibrotic collagenous tissue and mucinous degeneration in the right breast, consistent with the spectral CT findings. A comprehensive clinical timeline of this case is provided in **Table 1**.

**Table 1:** Clinical timeline of the case. This timeline summarizes key events from initial presentation through imaging, biopsy, surgery, and final pathological diagnosis. The multi-parametric DLCT analysis was performed retrospectively after surgery for research purposes.

Date	Event	Key findings
2025-04-27	Routine physical examination	Left breast lump detected
2025-04-30	Ultrasound	Bilateral BI-RADS 4 lesions (left: 4C; right: 4B)
2025-05.09	Ultrasound-guided biopsy of the bilateral breast lesions	Left: invasive carcinoma, grade II;Right:fibrous tissue hyperplasia
2025-05-12	Mammography	Left:breast carcinoma (BI-RADS 6);Right:hyperplasia of mammary glands (BI-RADS 2)
2025-05-13	Dual-layer detector spectralCT(Conventional CT analysis)	Left:poorly defined, mildly enhancing soft tissue nodule;Right:no enhancing lesion
2025-05-14	Contrast-enhanced breast MRI	Left:well-defined, homogeneously enhancing mass (BI-RADS 6);Right:no enhancing lesion
2025-05-15	Surgical Resection and Postoperative Pathology	Left:invasive ductal carcinoma with invasive ductal carcinoma *in situ* and lobular carcinoma *in situ* in the left breast;Right:extensive fibrotic collagenous tissue and mucinous degeneration
2025-05-21	Post-surgical follow-up	Uneventful recovery, discharged
2025-11-18	Retrospective DLCT multi-parametric analysis	Left: malignant features; Right: benign features (consistent with postoperative pathology)

## Discussion and conclusion

The incidence of breast cancer is increasing year by year, with a trend toward younger age at diagnosis. Currently, the main imaging modalities used for early diagnosis of breast lesions include ultrasound, mammography (X-ray), and magnetic resonance imaging (MRI). In China, ultrasound assessment is conducted according to the BI-RADS guidelines, which primarily classify lesions based on their morphological characteristics. However, BI-RADS 4 lesions present complex features on ultrasound images, with overlapping benign and malignant characteristics, making classification to some extent reliant on the subjective judgment of radiologists (7). Mammography is sensitive to microcalcifications and irregular high-density nodules but its diagnostic efficacy can be affected by factors such as glandular tissue density (8). Dynamic contrast-enhanced magnetic resonance imaging (DCE-MRI) diagnoses lesions based on their blood flow characteristics, but some BI-RADS 4 lesions exhibit atypical blood flow patterns, limiting its diagnostic effectiveness. Additionally, due to the longer examination time and higher cost of MRI, it is not easily applicable as a routine screening tool (9). Conventional CT, due to its low soft tissue contrast, has limited diagnostic value in breast lesions. In this case, the patient had lesions in both breasts, and the results from multiple imaging modalities, including ultrasound, mammography, MRI, and CT, were inconsistent. For the left breast lesion, all modalities indicated malignancy, but for the right breast lesion, ultrasound classified it as BI-RADS 4 (malignant), while mammography classified it as BI-RADS 2 (benign). No abnormalities were detected on MRI and conventional CT for the right lesion. This inconsistency in multi-modal imaging diagnoses highlights the limitations of traditional imaging techniques.

Based on the concept that biomedical imaging reflects underlying pathophysiological characteristics, quantitative image analysis can reveal intrinsic associations between these features. The multi-parametric information provided by spectral CT thus offers significant diagnostic value for the non-invasive differentiation of breast lesions. Conventional CT imaging utilizes polychromatic X-rays, generating images that reflect the average attenuation of tissues across a broad energy spectrum. In contrast, spectral CT separates polychromatic energy, enabling the reconstruction of virtual monochromatic images (VMIs) across a continuous energy range (40–200 keV) from a single scan. Low-keV VMI significantly enhances the contrast of iodinated contrast agents, thereby improving the conspicuity and detectability of iodine-avid lesions (10). Taihei Inoue et al (11). reported that, in patients with breast cancer, 40 keV VMI provided superior image quality and significantly improved lesion conspicuity compared with conventional CT images. In the present study, the left breast lesion appeared as a mildly enhancing, ill-defined mass on conventional venous-phase CT images, whereas no definite mass was identified in the right breast. In contrast, at 40 keV, the left breast lesion demonstrated focal, marked enhancement with well-delineated margins, and subtle enhancement was observed in the right breast. These findings underscore the added value of low-keV imaging for improving lesion detectability.

The attenuation values obtained across all monoenergetic levels can be connected to generate a characteristic spectral curve. The slope of this curve is closely associated with tissue composition. Different tissues or structures exhibit distinct curve slopes, whereas identical or similar tissues display comparable slopes (12).As demonstrated by Wang et al. (5), malignant tumors exhibit higher λHu. In our case, the spectral curve of the malignant left breast lesion exhibited a steep descending slope, whereas that of the benign right breast lesion was flat and gradual, running approximately parallel to the curve of normal glandular tissue. This distinct difference provided a crucial basis for the differential diagnosis.

Iodine density directly reflects the uptake of contrast agent by the lesion and serves as an indirect indicator of tumor vascularity. Malignant tissues typically exhibit stronger iodine uptake due to increased angiogenesis. In this case, the markedly higher iodine concentration in the left lesion substantially exceeded the optimal malignant threshold established by Demirler et al. (4), while the benign lesion’s density fell well below it (as detailed in the Case Presentation).

The effective atomic number represents the average atomic number of tissues or lesions, reflecting their intrinsic material composition (13). Jiang et al. (14) suggested that malignant nodules typically have higher cell density than benign nodules, which may result in higher Zeff values. In this case, the Zeff of the malignant lesion in the left breast, markedly higher than that of the benign lesion in the right breast. This finding is consistent with the aforementioned hypothesis.

The tumor microenvironment significantly affects tumor biological behavior; stroma-rich tumors are generally associated with poorer prognosis. ECV can non-invasively quantify tumor microenvironment characteristics, with higher values indicating a rich stroma and high microvascular density, associated with malignant biological behavior (15). Jiang et al. (6) demonstrated significant ECV differences between lung cancer and benign lung lesions (P < 0.001), with ECV exhibiting the optimal diagnostic performance among spectral CT parameters. In the present case, the marked difference in ECV between the malignant lesion (57%) and the benign lesion (11.8%) further supports the value of this parameter in characterizing breast lesions. This finding offers a promising direction for tumor microenvironment imaging biomarker research.

In conclusion, this case demonstrates that DLCT, in cases of inconsistent conventional imaging evaluation for BI-RADS 4 lesions, not only enhances lesion detection but also provides precise “qualitative” differentiation through its unique multi-parametric quantitative analysis. It should be emphasized that spectral CT is not recommended for routine breast cancer screening, given the associated radiation exposure and the potential for adverse reactions to CT contrast agents. In future clinical practice, DLCT could serve as an effective supplementary examination, and the quantitative information it provides preoperatively could help avoid diagnostic surgical biopsies for some patients, thus enabling more precise personalized management.

This study has certain limitations: as a single-center retrospective case report, it provides valuable insights, but the results still need to be further validated in prospective, large-sample studies.

## Data Availability

The original contributions presented in the study are included in the article/supplementary material. Further inquiries can be directed to the corresponding author.
